# Anti-Inflammatory Effects Induced by a Polyphenolic Granular Complex from Olive (*Olea europaea*, Mainly *Cultivar coratina*): Results from In Vivo and Ex Vivo Studies in a Model of Inflammation and MIA-Induced Osteoarthritis

**DOI:** 10.3390/nu14071487

**Published:** 2022-04-02

**Authors:** Lucia Recinella, Laura Micheli, Annalisa Chiavaroli, Maria Loreta Libero, Giustino Orlando, Luigi Menghini, Alessandra Acquaviva, Simonetta Di Simone, Claudio Ferrante, Carla Ghelardini, Luigi Brunetti, Sheila Leone

**Affiliations:** 1 Department of Pharmacy, “G. d’Annunzio” University, 66013 Chieti, Italy; lucia.recinella@unich.it (L.R.); annalisa.chiavaroli@unich.it (A.C.); maria.libero@unich.it (M.L.L.); giustino.orlando@unich.it (G.O.); luigi.menghini@unich.it (L.M.); alessandra.acquaviva@unich.it (A.A.); simonetta.disimone@unich.it (S.D.S.); claudio.ferrante@unich.it (C.F.); sheila.leone@unich.it (S.L.); 2 Department of Neuroscience, Psychology, Drug Research and Child Health-NEUROFARBA-Pharmacology and Toxicology Section, University of Florence, 50139 Florence, Italy; laura.micheli@unifi.it (L.M.); carla.ghelardini@unifi.it (C.G.)

**Keywords:** *Cultivar coratina*, inflammation, osteoarthritis, hydroxytyrosol, pain

## Abstract

MOMAST^®^ GR25 is a polyphenolic granular complex from olive pressing juice with high total content in polyphenols. In this work, we evaluated the possible anti-inflammatory effects of MOMAST^®^ GR25 in both acute and chronic inflammatory models. MOMAST^®^ GR25 decreased the levels of prostaglandin (PG) E_2_ and 8-iso-PGF_2α_ in isolated rat colon, liver, and heart specimens stimulated with lipopolysaccharide (LPS). In vivo, compared to controls, rats treated with MOMAST^®^ GR25 (100 mg/kg to 1 g/kg) showed a significant reduction in both licking/biting time in the formalin test. In a rat model of osteoarthritis by monoiodoacetate (MIA) injection, MOMAST^®^ GR25 showed pain-relieving properties when acutely administered, reducing mechanical hyperalgesia and spontaneous pain. Moreover, a repeated daily treatment with MOMAST^®^ GR25 (300 mg/kg) fully counteracted osteoarticular pain without the development of tolerance to the antinociceptive effect. Taken together, our present findings showed that MOMAST^®^ GR25 could represent a potential strategy for the treatment of inflammation and pain.

## 1. Introduction

*Olea europaea* L. (family Oleaceae) is a typical plant of the Mediterranean area known for its recognized pharmacological properties [1]. The beneficial effects of *Olea europaea* have been attributed to the presence of phenolic components, which showed antioxidant, anti-inflammatory, immunomodulatory, anti-atherogenic, hepato-protective, anticancer, antimicrobial, hypolipidemic, and hypoglycemic activities [2]. In particular, the olive tree polyphenols activated endogenous antioxidant enzymes, such as glutathione S-transferase and glutathione peroxidase, as well as scavenged free radicals and reactive oxygen species (ROS) [3,4,5].

Hydroxytyrosol (HT) and the secoiridoid oleuropein (OE) are two abundant phenolic compounds in virgin oil, olives, and waste water from olive oil production [6,7,8]. Particularly, HT were found to be able to exert antioxidant and scavenging activities comparable to OE and catechol [9]. A wide body of evidence suggested that HT exerted anti-atherogenic, anti-inflammatory, as well as anti-thrombotic activities. In addition, it counteracted a number of chronic diseases, including insulin resistance, hyperglycemia, and metabolic syndrome, as well as improved endothelial function [10]. Many studies demonstrated that OE induced protective effects in multiple tissues, including the heart, central nervous system, liver, and stomach, which could be related to its antioxidant and anti-inflammatory properties [11].

Interestingly, verbascoside is a phenylpropanoid glycoside found in a variety of plant species [12,13], including olive. It has been shown to exert anti-inflammatory properties, by decreasing levels of various pro-inflammatory markers [14,15]. The potential role of verbascoside as a possible food antioxidant has also been suggested by Cardinali and collaborators (2012) [16]. Finally, various studies suggested multiple biological activities induced by tyrosol, such as antioxidant, anticancer, anti-inflammatory, along with cardioprotective and neuroprotective effects [17]. It has also been suggested that a key role of tyrosol is diabetes mellitus management, which could be related to its anti-inflammatory effects [18].

MOMAST^®^ GR25 (Bioenutra, Ginosa, Italy) is a polyphenolic granular complex rich in HT and other polyphenols from olive (*Olea europaea,* mainly *Cultivar coratina*) (production area: Puglia, Italy). MOMAST^®^ GR25 is characterized by the presence of HT, tyrosol, verbascoside, OE, and other polyphenols, whose identification was performed according to the laboratory organization and technical conditions of analysis requirements [19] (Table 1).

A previous study from ours showed that MOMAST^®^ HY100 and MOMAST^®^ HP30 (Bioenutra, Ginosa, Italy), two polyphenolic liquid complexes from olive (*Olea europaea,* mainly *Cultivar coratina*) pressing juice, exerted protective effects, by decreasing lactate dehydrogenase (LDH), prostaglandin (PG)E_2_, and 8-iso-PGF_2α_, levels, as well as tumor necrosis factor α (TNFα), cyclooxygenase (COX)-2, and inducible nitric oxide synthase (iNOS) gene expression, in rat isolated tissues, after treatment with lipopolysaccharide (LPS) [20].

In the present study, we evaluated the possible anti-inflammatory effects of MOMAST^®^ GR25 on isolated rat colon, liver, and heart specimens treated with LPS, a validated ex vivo model of inflammation, comparing it to HT. Moreover, we studied the acute and chronic effects of MOMAST^®^ GR25 in a formalin test and in a rat model of monoiodoacetate (MIA)-induced osteoarthritis.

## 2. Materials and Methods

Adult Sprague-Dawley male rats (200–250 g) were housed in Plexiglass cages (40 cm × 25 cm × 15 cm), in colony rooms (22 ± 1 °C; 60% humidity), on a 12 h/12 h light/dark cycle (light phase: 07:00–19:00 h), with free access to tap water and food, 24 h/day throughout the study, with no fasting periods. Rats were fed with a standard laboratory diet. The detailed Materials and Methods are provided in Supplementary File S1.

Colon, liver, and heart specimens were obtained from rats treated with the vehicle from our previous experiments, approved by Local Ethical Committee (University “G. d’Annunzio” of Chieti-Pescara) and Italian Health Ministry (Italian Health Ministry authorization N. 880, delivered on 24 August 2015).

All animal manipulations were carried out according to the Directive 2010/63/EU of the European parliament and of the European Union council (22 September 2010) on the protection of animals used for scientific purposes and with IASP. The ethical policy of the University of Florence complies with the Guide for the Care and Use of Laboratory Animals of the US National Institutes of Health (NIH Publication No. 85–23, revised 1996; University of Florence assurance number: A5278-01). Formal approval to conduct the manipulations and experiments described was obtained from the Italian Ministry of Health (No. 171/2018-PR) and from the Animal Subjects Review Board of the University of Florence. 

### 2.1. Ex Vivo Studies

Rats were sacrificed by CO_2_ inhalation (100% CO_2_ at a flow rate of 20% of the chamber volume per min), and tissue specimens were immediately collected and maintained in a humidified incubator with 5% CO_2_ at 37 °C for 4 h, in RPMI buffer with added bacterial LPS (10 μg/mL) (incubation period).

During the incubation period, tissues were treated with scalar concentrations of MOMAST^®^ GR25 (5 and 25 µg/mL) and HT (5 and 25 µg/mL), which were used as the reference standard. Tissue supernatants were collected, and the PGE_2_ and 8-iso-PGF_2α_ levels (ng/mg wet tissue) were measured by radioimmunoassay (RIA), as previously reported [20].

### 2.2. In Vivo Studies

After 2-week acclimation, MOMAST^®^ GR25 was orally administered, suspended in a 1% carboxymethylcellulose sodium salt (CMC) solution, at doses of 100 mg/kg, 300 mg/kg and 1g/kg. A standardization of testing conditions was carefully taken [21].

### 2.3. Formalin Test

Formalin solution was injected, subcutaneously, under the plantar surface of a hind paw, and pain-related behaviors were scored during two successive phases, as previously reported [21].

### 2.4. Monoiodoacetate (MIA)-Induced Osteoarthritis

A MIA (Sigma-Aldrich, Milan, Italy) injection was used to induce unilateral osteoarthritis into the tibio-tarsal joint [22,23]. A monolateral injection of MIA (2 mg/25 μL of saline) was performed on the ipsilateral paw. Control rats were injected with saline solution.

### 2.5. Paw Pressure Test

The evaluation of mechanical hyperalgesia was performed by an analgesimeter (Ugo Basile, Varese, Italy), as previously described by Leighton and collaborators (1988) and Bird and collaborators (2016) [24,25].

### 2.6. Incapacitance Test

The evaluation of spontaneous pain was performed by an incapacitance apparatus (Linton Instrumentation, Norfolk, UK) in order to record variations in the postural equilibrium [26,27]. An unequal distribution of weight on hind limbs displayed a monolateral decreased pain threshold [22].

### 2.7. Statistical Analysis

Statistical analysis was performed using GraphPad Prism version 5.01 for Windows (GraphPad Software, San Diego, CA, USA). Means ± SEM were measured for each experimental group and statistical analysis of data was performed by one-way analysis of variance (ANOVA), followed by Newman–Keuls comparison multiple test or by Bonferroni test [28].

## 3. Results

### 3.1. Inhibitory Effects of MOMAST^®^ GR25 (5 and 25 µg/mL) on LPS-Induced PGE_2_ and 8-iso-PGF_2α_ Levels in Colon, Liver and Heart Specimens

**Figure 1:**
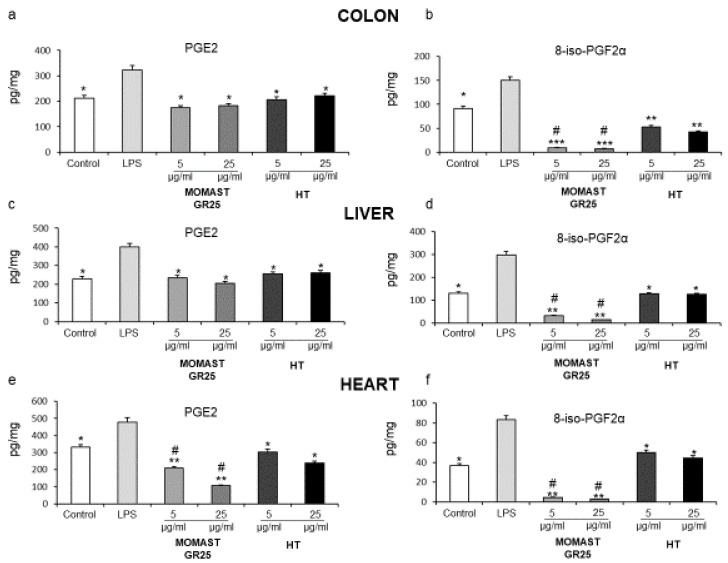
Effects of MOMAST^®^ GR25 (5 and 25 µg/mL) on prostaglandin (PG)E_2_ and 8-iso-PGF_2α_ levels (pg/mg wet tissue) in rat colon (**a**,**b**), liver (**c**,**d**), and heart (**e**,**f**) specimens. Data were reported as means ± SEM. One-way analysis of variance (ANOVA), *p* < 0.01; *post-hoc* test, * *p* < 0.05, ** *p* < 0.01 and *** *p* < 0.005 vs. LPS-treated group: ^#^ *p* < 0.005 vs. HT group.

We evaluated the modulatory effects of MOMAST^®^ GR25 (5 and 25 µg/mL) supplementation on PGE_2_ and iso-PGF_2α_ levels in colon, liver, and heart treated with LPS. In this context, PGE_2_ and 8-iso-PGF_2α_ levels, in the supernatants of tissues, were analyzed by RIA, following the treatment of tissue specimens with LPS+MOMAST^®^ GR25 (5 and 25 µg/mL), LPS+HT (5 and 25 µg/mL), LPS, or vehicle. LPS treatment significantly increased PGE_2_ and 8-iso-PGF_2α_ levels, with respect to vehicle, in all tested tissues. Compared to LPS, both MOMAST^®^ GR25 (5 and 25 µg/mL) and HT (5 and 25 µg/mL) decreased PGE_2_ and 8-iso-PGF_2α_ levels induced by LPS, in colon, liver and heart specimens (Figure 1a–f).

However, MOMAST^®^ GR25 (5 and 25 µg/mL) was more effective in inhibiting LPS-induced 8-iso-PGF_2α_ in all tissues analyzed, with respect to HT-treated groups (Figure 1b,d,f). In addition, in heart specimens, MOMAST^®^ GR25 (5 and 25 µg/mL) was able to significantly reduce PGE_2_ levels as compared to HT (Figure 1e).

### 3.2. MOMAST^®^ GR25 (100, 300 mg/kg and 1 g/kg) Reduced Responsiveness to Acute Inflammatory Stimuli

**Figure 2:**
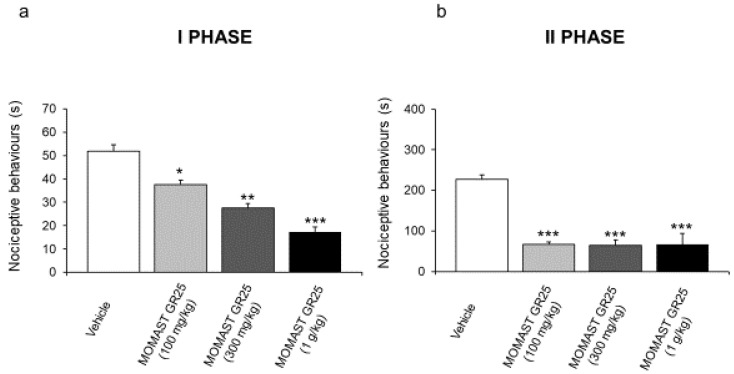
Effect of MOMAST^®^ GR25 (100, 300 mg/kg, and 1 g/kg) oral administration on the acute nociceptive response in the formalin test (n = 6 for each group of treatment). The nociceptive behavior time (seconds) of licking, shaking, and biting was measured during the period of 0–5 min (first phase) (**a**) and 20–60 min (second phase) (**b**). Data were reported as means ± SEM. One-way analysis of variance (ANOVA), *p* < 0.01; *post-hoc* test, * *p* < 0.05, ** *p* < 0.01, and *** *p* < 0.005 vs. vehicle-treated animals.

Osteoarthritis is strongly related to inflammation, a response evoked by the mechanical damage of joints after the reduction in cartilage. Starting from this, a painful disease characterized by loss of function evolves [29]. For this reason we preliminarily evaluated the efficacy of the extract in a model of inflammation. Aiming to evaluate the responsiveness to acute inflammatory stimuli, rats were tested by a formalin test. MOMAST^®^ GR25 (100, 300 mg/kg, and 1g/kg) was orally administered 20 min before the test. Control animals received vehicle (1% CMC). After intraplantar injection of formalin, rats treated with MOMAST^®^ GR25 (100, 300 mg/kg, and 1g/kg) showed short nociceptive behavioral responses (first and second phase) compared to vehicle-treated group (Figure 2). In particular, both in the first and second phase, we showed that oral administration of MOMAST^®^ GR25 (100, 300 mg/kg, and 1 g/kg) significantly decreased nociceptive behavioral response compared to the vehicle-treated group (Figure 2a,b). In particular, the reduction in nociceptive behavioral response, induced by MOMAST^®^ GR25, was found to be dose-dependent in the first phase of the formalin test (Figure 2a).

### 3.3. Effects of Acute Administration of MOMAST^®^ GR25 (100, 300 mg/kg and 1 g/kg) in Experimental Osteoarthritis

To confirm the potential anti-inflammatory effect of MOMAST^®^ GR25, we used a model of MIA-induced osteoarthritis. Behavioral assessments were carried out from the 14th day after the induction of damage (obtained by intraarticular administration of MIA) when the neuropathic component is predominant. The animals were tested before (0 min) and after the administration of MOMAST^®^ GR25. The effect of MOMAST^®^ GR25 on the animal’s pain threshold was measured using a painful mechanical stimulus (Paw Pressure test), while the incapacitance test was used to measure spontaneous pain by evaluating the difference between the weight burdened on the contralateral paw vs the ipsilateral one. In the presence of monolateral pain, the animal tends to load more weight on the contralateral paw than on the ipsilateral leg injected with algogen MIA agent.

**Figure 3:**
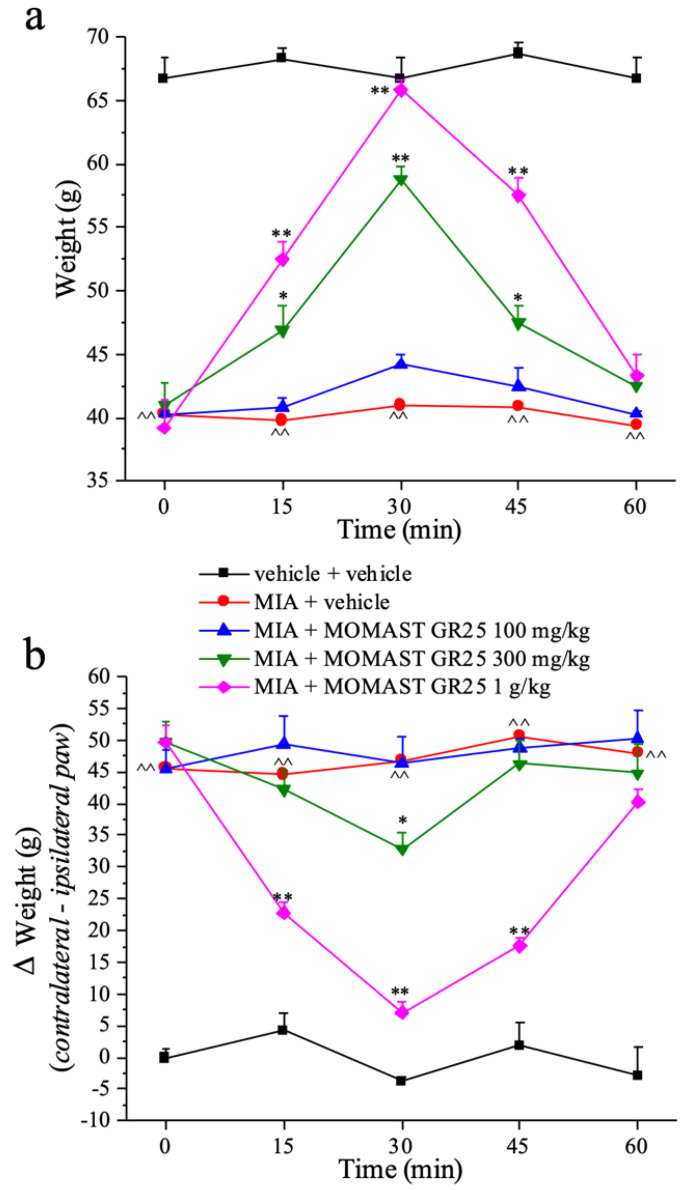
Effect of the acute administration of MOMAST^®^ GR25 on monoiodoacetate (MIA)-induced osteoarthritis in the rat, (**a**) Paw pressure test; (**b**) Incapacitance test. Osteoarthritis was induced by injection of MIA (2 mg/25 µL) into the tibio-tarsal joint. Fourteen days after MIA injection, MOMAST^®^ GR25 (100 mg/kg–1 g/kg) was suspended in 1% carboxymethylcellulose sodium salt (CMC) and orally administered. Tests were performed before and after treatment. Each value represents the mean of six rats performed in two different experimental sets. One-way analysis of variance (ANOVA), *p* < 0.05; *post-hoc* test, ^^ *p* < 0.01 vs vehicle + vehicle treated animals; * *p* < 0.05, and ** *p* < 0.01 vs MIA + vehicle treated animals.

Figure 3a displays the effect of the acute administration of MOMAST^®^ GR25, at doses of 100 mg/kg, 300 mg/kg, and 1 g/kg, 14 days after damage induced by MIA on the ipsilateral paw. The weight borne by these animals on the ipsilateral paw at the paw pressure test is significantly reduced compared to control animals (vehicle + vehicle) (43 ± 0.8 g vs. 66.7 ± 1.7 g, respectively). In this pain model, the highest tested dose (1 g/kg) of MOMAST^®^ GR25 completely reversed the hyperalgesia induced by MIA at 30 min after administration, reaching a value of 65.8 ± 0.8 g, and the anti-hyperalgesic effect was still active even at 15 min (52.5 ± 1.4 g) and remained up to 45 min (57.5 ± 1.4 g). The intermediate dose of 300 mg/kg increased the threshold of pain in mice from 15 min from administration up to 45 min. The anti-hyperalgesic effect generated is lower than that achieved with the dose of 1 g/kg but statistically significant with respect to the control. The dose of 100 mg/kg showed a mild effect, increasing the pain threshold only 30 min after treatment (44.2 ± 0.8 g). No significant change was found on the contralateral paw after administration of MOMAST^®^ GR25, thus excluding any central analgesic effect (Table 2).

Figure 3b shows the incapacitance test evaluating the spontaneous pain of the animal by the weight that is burdened on the two hind legs. The intra-articular injection of MIA resulted in a postural imbalance translated into an increased weight burdened on the contralateral paw, compared to the ipsilateral one, and reported in the Figure 3b as delta (weight). The values recorded in this group exceeded 40 g compared to the values close to zero found in the control group (vehicle + vehicle). The maximum dose of MOMAST^®^ GR25 (1 g/kg) was able to reduce the weight difference burdened by the animal on the hind legs 15 min after administration (22.8 ± 1.6 g), the efficacy increased at 30 min, reaching the value of 7.1 ± 1.8 g, remained significant even at 45 min (17.6 ± 1.1 g), and then ended at 60 min (40.3 ± 2.0 g). The intermediate dose of 300 mg/kg partially reduced the weight difference between the two legs at 30 min after treatment, while the lowest dose in this test was ineffective (Figure 3b).

### 3.4. Effect of Repeated Administration of MOMAST^®^ GR25 (100, 300 mg/kg and 1 g/kg) in an Animal Model of Osteoarthritis

The same behavioral measures (paw pressure and incapacitance tests) were performed following repeated treatments with MOMAST^®^ GR25, dosed at 300 mg/kg (administered twice daily for 14 consecutive days). Using this experimental protocol, the protective effect of MOMAST^®^ GR25 on MIA-induced osteoarthritic damage was evaluated. Treatment was administered daily and started in parallel with the induction of the damage (day 1). Pain measurements were conducted 7 and 14 days after the beginning of the experiment, both 24 h after the last administration and 30 min after the last daily treatment.

**Figure 4:**
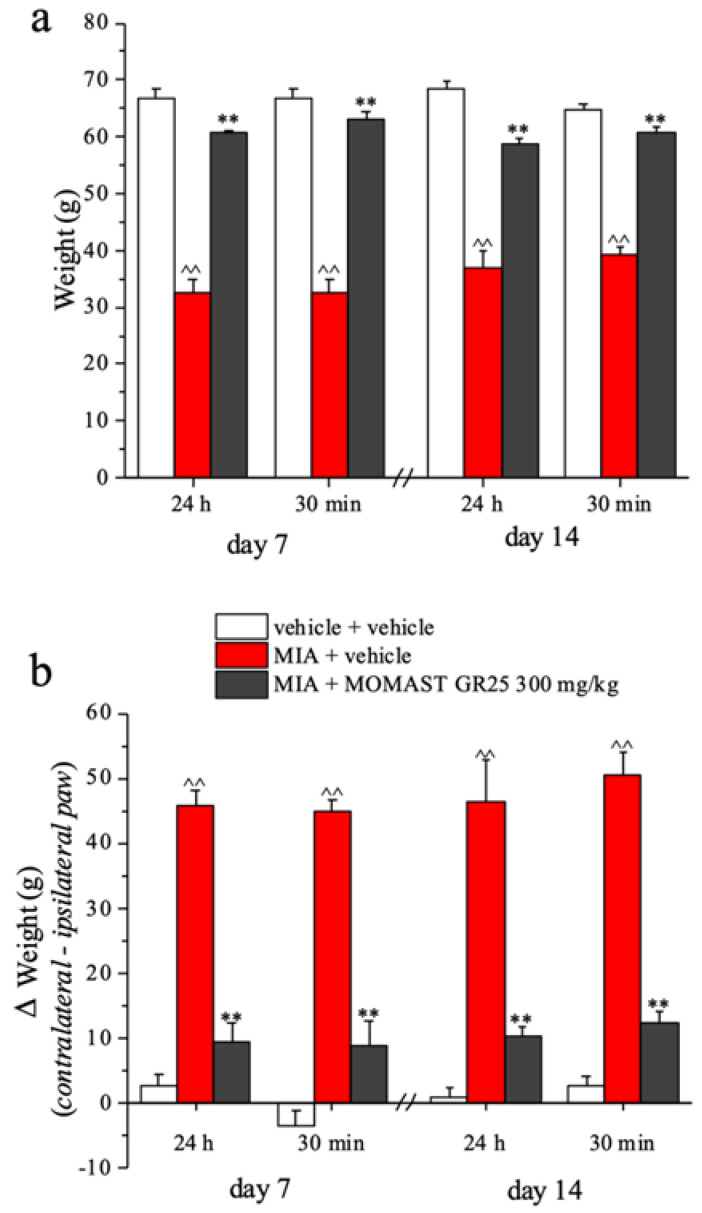
Effect of repeated administration of MOMAST^®^ GR25 on monoiodoacetate (MIA)-induced osteoarthritis, (**a**) paw pressure test; (**b**) incapacitance test. Osteoarthritis was induced by the injection of MIA (2 mg/25 µL) into the tibio-tarsal joint. Starting from MIA injection (day 1), MOMAST^®^ GR25 300 mg/kg was suspended in 1% carboxymethylcellulose sodium salt (CMC) and orally administered twice daily. Behavioral measurements were performed 24 h and 30 min after administration on days 7 and 14. Each value represents the mean of six rats performed in two different experimental sets. One-way analysis of variance (ANOVA), *p* < 0.05; *post-hoc* test, ^^ *p* < 0.01 vs vehicle + vehicle treated animals; ** *p* < 0.01 vs MIA + vehicle treated animals.

Figure 4a shows the effectiveness of the treatment in reducing mechanical hyperalgesia in the paw pressure test. At day 7, animals injected with MIA (MIA + vehicle) show a significant reduction in the algic threshold compared to the control group (vehicle + vehicle) (32.5 ± 2.5 g vs. 66.7 ± 1.7 g, respectively). Daily treatment with MOMAST^®^ GR25 prevented the development of joint pain when it was measured 24 h after the last dose; the measurement made 30 min following the new daily administration showed no significant changes in the algic threshold value recorded. The anti-hyperalgesic effect did not show tolerance since similar results were obtained 14 days after the start of treatment. At this time, MOMAST^®^ GR25 was able to prevent the onset of mechanical hyperalgesia measured with the paw pressure test. Similar results were found in the incapacitance test (Figure 4b). Daily treatment with MOMAST^®^ GR25 significantly prevented postural imbalance induced by damage with the algogen agent MIA. The effect is similar both 7 days and 14 days after the start of the experiment and without significant changes between the measurements made 24 h or 30 min after each daily treatment.

## 4. Discussion

Our study demonstrates that MOMAST^®^ GR25 and HT modulated the inflammatory pathways and oxidative stress in all isolated tissues challenged with LPS. Interestingly, MOMAST^®^ GR25 was more effective in reducing LPS-induced 8-iso-PGF_2α_ in all evaluated tissues. Actually, free radical-reducing and scavenging properties of olive polyphenols, including OE and HT, could be involved, at least in part, in these effects [30,31]. In this context, Carrasco-Pancorbo and collaborators (2005) classified HT, deacetoxy OE aglycon, and OE aglycon as the strongest antioxidants in virgin olive oils [32]. Oxidative stress is characterized by an imbalance in the homeostasis of pro-oxidant/antioxidant, and increased production of reactive oxygen/nitrogen species, as well as free radicals, can lead to peroxidation reactions on various biomolecules such as nucleic acids, proteins, and lipids [33,34]. Interestingly, verbascoside was shown to induce a protective effect on human cells, which could be related to its direct antioxidant and free radical scavenging properties, as well as to the up-regulation of endogenous detoxifying systems [35]. Actually, we can speculate that the protective effects exerted by MOMAST^®^ GR25 could be related, albeit partially, to the presence of verbascoside. Additionally, a wide body of evidence suggested a critical role played by oxidative damage in the pathogenesis of multiple chronic diseases, such as chronic inflammation, cancer, and diabetes [36,37,38]. Acute administration MOMAST^®^ GR25 decreased in a dose-dependent manner the responsiveness to acute chemical inflammatory stimuli, in mice. In agreement, an extract from olive leaf enriched in HT inhibited hyperalgesia and inflammatory swelling, and decreased gene expression of proinflammatory cytokine in a rodent model of acute inflammation and pain induced by carrageenan [39]. In addition, verbascoside has been found to exert antinociceptive and anti-inflammatory activities [40], as well as to suppress PGE_2_ production. In addition, it exerted protective effects against oxygen free radical-induced peroxidative damage in biological membranes [41]. On the basis of these data, we performed our study on a rat model of MIA-induced osteoarthritis. This model is easy to perform in rodents and is able to reproduce osteoarthritis-like lesions and functional impairment resembling those seen clinically [42,43]. MIA inhibits glyceraldehyde-3-phosphatase activity [44,45]; its intra-articular injection elicits transient inflammation and causes chondrocyte cell death, leading to synovial thickening, formation of osteophytes, loss of cartilage, and cartilage fibrillation [42,46,47], in the presence of a crucial component of damage induced by oxidative stress [48]. All these factors determine persistent inflammatory pain, led to significant alterations in hind limb weight bearing and referred mechanical hypersensitivity development, starting from the 14th day after damage, which also includes a neuropathic component [29]. Indeed, nonsteroidal anti-inflammatory drugs are useful during the first inflammatory phase of MIA-induced osteoarthritis but loose efficacy during the following neuropathic phase when medications such as gabapentin are effective [29]. Our findings showed that MOMAST^®^ GR25 exerted therapeutic and protective properties on rat model of MIA-induced osteoarthritis. A potential limitation of our study is that we have not performed histological analysis, which could represent an important aspect of this research. On the other hand, Horcajada and collaborators (2015) showed that OE significantly slowed down the progression of osteoarthritis lesions in animals [49]. In particular, these results were confirmed by histological analysis, showing a decreased cartilage degradation score and synovial histological score, with respect to the control [49]. In this model, MOMAST^®^ GR25, acutely administered before the behavioral tests 14 days after damage, determined pain relief and a reduction in postural unbalance in a dose-dependent manner (100 mg/kg–1 g/kg) for at least 45 min. The therapeutic effect was accompanied by the protective one when the treatment with MOMAST^®^ GR25 (300 mg/kg) started the same day of MIA injection (day 1) and continued, daily, for two weeks. Chronic treatment with MOMAST^®^ GR25 fully prevented the development of mechanical hyperalgesia and spontaneous pain at both 7 and 14 days. Interestingly, the repeated administration of MOMAST^®^ GR25 did not induce tolerance to the anti-nociceptive effects, in contrast to other drugs, such as morphine, that shows tolerance to the analgesic effects both in naïve [50,51] and in MIA-treated animals [52], which represents one of the most important side effects for the use of opioids in chronic persistent pain [53]. Furthermore, MOMAST^®^ GR25 did not significantly modify the pain threshold of the contralateral (control) paw, thus excluding central analgesic properties and highlighting a pure anti-hypersensitive profile. Many of the behavioral effects are in line with the antioxidant property of MOMAST^®^ GR25 previously described. Mounting evidence suggests that the production of ROS is upregulated during persistent inflammatory and neuropathic pain [54]. To support these findings, Wang and colleagues (2004) demonstrated that superoxide, a type of ROS, is involved in the modulation of pain that accompanies inflammation and highlighted that a synthetic compound, which mimics the enzymatic function of superoxide dismutase (SOD), was able to prevent the development of inflammatory processes, as well as hyperalgesia, following injection of an inflammatory agent into the rat paw [55]. Olive oil and polyphenols derived from olive, including HT, tyrosol, and OE, could represent potential candidates for the treatment of osteoarthritis. In this context, HT has been evaluated in osteoarthritis models with interesting results [56]. In this scenario, HT can be considered the most important player in the protective role of MOMAST^®^ GR25 in osteoarthritis pathology since it has been able to exert direct effects on the antioxidant system by stimulating glutathione peroxidase, SOD, and glutathione reductase activities and also maintaining higher reduced glutathione (GSH) levels in cells [57,58]. In addition, the marked anti-inflammatory effects of MOMAST^®^ GR25 could also be attributed to presence of verbascoside. It possesses substantial beneficial activities and exerts anti-inflammatory, analgesic, and antioxidative properties in in vitro and in vivo studies [59,60,61]. In preclinical studies, verbascoside was able to inhibit levels of Bax, Bcl-2, nuclear factor-κB, iNOS, and myeloperoxidase, showing anti-inflammatory benefits in a model of periodontitis [60]. Additionally, this compound inhibits mechanical pain in two different animal models of hyperalgesia [62]. Doses for acute treatment were established on the basis of published data [63]. The experimental evidence obtained with the acute protocol informed the second phase experiments in which repeated daily administration with the optimal dose of 300 mg/kg was performed. The dosages proposed in the present research are useful for a clinical translation. On the basis of well-known metabolic differences among rodents and humans, the doses administered in mice or rats as mg/kg body weight can be converted, in the equivalent dose, in mg in humans, by using a ratio ranging between 1:1 and 1:5–7 in relation to the type of compound and its metabolism [64]. Generally, tables [65,66] exist indicating the conversion of mg/kg mouse in mg in humans is as follows using specific factors: /kg mouse /12.3) × 70 = 569 mg/human; so the proposed dose of 300 mg/kg could be about 1.5 g/human.

Kim et al. (2016) showed that serum PGE2 levels were significantly higher in the vehicle-treated MIA group compared with the control (treated with normal saline, but not MIA) group, in rats, suggesting a possible link between behavioral measures and PGE2 levels [67]. Accordingly, decreased serum levels of PGE2 induced by OE was hypothesized to be related to its protective effects in osteoarthritis physiopathologic processes [49]. Overproduction of PGE2 was shown to be highly associated with overexpression of pro-inflammatory cytokines including, inducible nitric oxide synthase (iNOS) and cyclooxigenase (COX)-2 in inflammatory responses [68] On the basis of these and our previous [20] findings, we can speculate that MOMAST^®^ GR25 could reduce production of these pro-inflammatory markers in serum. Another limitation of our study is that we have not evaluated the effects of the MOMAST^®^ GR25 administration on systemic cytokine levels, which could contribute to further deepening the potential mechanisms involved in the protective effects induced by this polyphenolic complex. In conclusion, taken together, our present findings showed that MOMAST^®^ GR25 could represent a potential strategy for the treatment of inflammatory reactions and pain.

## Figures and Tables

**Table 1:** Characteristics of the Polyphenolic Complex MOMAST^®^ GR25.

Name:	MOMAST^®^ GR25
Description:	Polyphenolic active complex of hydroxytyrosol-(*Olea europaea* fruit extract) with total polyphenolic content of 25 g/kg
Source Type:	Mainly *Cultivar coratina*
Physical State:	Granular
Appearance:	Powder-light beige
Moisture:	N. A.
Ash:	Less than 10% (600 °C)
Total heavy metals (as Pb):	Less than 10 ppm
Total Plate Count:	Less than 100 cfu/g
Pesticides:	Absence
**POLYPHENOLIC CONTENT**	
Hydroxytyrosol (HPLC)	1.0–2.5%
Verbascoside (HPLC)	0.02–0.5%
Tyrosol (HPLC)	0.1–1.0%
Oleuropein (HPLC)	<0.5%
Total Polyphenols (HPLC)	>2.5%

**Table 2:** Effect of acute administration of MOMAST^®^ GR25 on MIA-induced osteoarthritis: contralateral paw.

**Paw Pressure Test** Contralateral Paw Weight (g)					
Treatments	0 min	15 min	30 min	45 min	60 min
vehicle + vehicle	70.3 ± 1.7	65.8 ± 1.5	66.7 ± 1.7	67.3 ± 1.6	68.3 ± 1.7
MIA + vehicle	68.3 ± 0.8	67.0 ± 0.9	69.2 ± 0.8	70.1 ± 0.5	64.0 ± 1.3
MIA + MOMAST^®^ GR25 100 mg/kg	66.7 ± 1.7	68.3 ± 1.7	68.3 ± 1.7	65.3 ± 0.3	65.2 ± 0.2
MIA + MOMAST^®^ GR25 300 mg/kg	66.9 ± 1.0	67.5 ± 1.3	66.9 ± 1.0	66.3 ± 1.0	65.4 ± 0.3
MIA + MOMAST^®^ GR25 1 g/kg	65.8 ± 0.8	66.7 ± 1.7	66.7 ± 1.7	66.7 ± 1.7	66.7 ± 1.7

Osteoarthritis was induced by injection of monoiodoacetate (MIA) (2 mg/25 µL) into the tibio-tarsal joint. Fourteen days after MIA injection, MOMAST^®^ GR25 was suspended in 1% carboxymethylcellulose sodium salt (CMC) and orally administered. Paw pressure test was performed before and after treatment. Each value represents the mean of six animals performed in two different experimental sets. One-way analysis of variance (ANOVA), followed by the Bonferroni test, revealed no significant differences.

## Data Availability

The data presented in this study are available on request from the corresponding author.

## Supplementary Materials

The following supporting information can be downloaded at: https://www.mdpi.com/article/10.3390/nu14071487/s1, File S1: Materials and Methods.
