# Supplementary material for: Anti-Inflammatory Effects Induced by a Polyphenolic Granular Complex from Olive (Olea europaea, Mainly Cultivar coratina): Results from In Vivo and Ex Vivo Studies in a Model of Inflammation and MIA-Induced Osteoarthritis
Source: Nutrients. 2022 Apr 2;14(7):1487. doi: 10.3390/nu14071487 (PMC9002755; doi:10.3390/nu14071487)
Supplement: Supplementary file 1 [file nutrients-14-01487-s001.zip › nutrients-1622492-supplementary.pdf]

Materials and Methods

Male adult Sprague-Dawley rats (200-250 g) were housed in Plexiglass cages (40 cm × 25 cm × 15 cm), two rats per cage, in climatized colony rooms (22 ± 1 °C; 60% humidity), on a 12 h/12 h light/dark cycle (light phase: 07:00 - 19:00 h), with free access to tap water and food, 24 h/day throughout the study, with no fasting periods. Rats were fed with a standard laboratory diet (3.5% fat, 63% carbohydrate, 14% protein, 19.5% other components without caloric value; 3.20 kcal/g).

Housing conditions and experimentation procedures were strictly in accordance with the European Union ethical regulations on the care of animals for scientific research.

According to the recognized ethical principles of "Replacement, Refinement and Reduction of Animals in Research", colon, liver and heart specimens were obtained as residual material from vehicle-treated rats randomized in previous experiments approved by Local Ethical Committee (University "G. d'Annunzio" of Chieti-Pescara) and Italian Health Ministry (Italian Health Ministry authorization N. 880, delivered on 24th August 2015). All animal manipulations were carried out according to the Directive 2010/63/EU of the European parliament and of the European Union council (22 September 2010) on the protection of animals used for scientific purposes and with IASP. The ethical policy of the University of Florence complies with the Guide for the Care and Use of Laboratory Animals of the US National Institutes of Health (NIH Publication No. 85-23, revised 1996; University of Florence assurance number: A5278-01). Formal approval to conduct the experiments described was obtained from the Italian Ministry of Health (No. 171/2018-PR) and from the Animal Subjects Review Board of the University of Florence.

2.1. *Ex vivo* studies

Rats were sacrificed by CO_2 inhalation (100% CO_2 at a flow rate of 20% of the chamber volume per min) and colon, liver and heart specimens were immediately collected and maintained in humidified incubator with 5% CO_2 at 37 °C for 4 h, in RPMI buffer with added bacterial LPS (10 μg/mL) (incubation period).

During the incubation period, tissues were treated with scalar concentrations of MOMAST(®) GR25 (5 and 25 μg/ml) and HT (5 and 25 μg/ml), used as the reference standard. Tissue supernatants were collected, and the PGE_2 and 8-iso-PGF_2 levels (ng/mg wet tissue) were measured by radioimmunoassay (RIA), as previously reported [1]. Briefly, specific anti-8-iso-$PGF_{2\alpha}$ and anti-PGE_2 were developed in the rabbit; the cross-reactivity against other prostanoids is <0.3%. One hundred microliters of prostaglandin standard or sample were incubated overnight at 4°C with the 3H-prostaglandin (3000 cpm/tube; NEN) and antibody (final dilution: 1:120 000; kindly provided by the late Prof. G. Ciabattoni), in a volume of 1.5 mL of 0.025 M phosphate buffer. Free and antibody-bound prostaglandins were separated by the addition of 100 μL 5% bovine serum albumin and 100 μL 3% charcoal suspension, followed by centrifuging for 10 min at 4000×g at 5°C and decanting off the supernatants into scintillation fluid (Ultima Gold™, Perkin Elmer) for β emission counting. The detection limit of the assay method is 0.6 pg/mL.

2.2. *In vivo* studies

After 2-week acclimation, MOMAST(®)GR25 was orally administered suspended in a 1% carboxymethylcellulose sodium salt (CMC) solution, at doses of 100 mg/kg, 300 mg/kg and 1g/kg. All solutions were freshly prepared before use. All treatments were administered at 09:00 a.m., and all *in vivo* tests were performed between 10:00 a.m. and 12:00 a.m. to minimize circadian variations in sensitivity to pain. Care was taken to standardize testing conditions [2]. All materials used for each test were thoroughly cleaned after test completion for each rat. At the end of each test, the animals were returned to their home cages, and the apparatus was cleaned with 75% ethanol and dried before the next procedure. Each test was conducted on the same group of animals (n=6 for each group of treatment).

2.3. Formalin test.

A diluted formalin solution was injected subcutaneously under the plantar surface of a hind paw, and pain related behaviors were scored during two successive phases [2]. The first phase (0–5 min.) reflects direct activation of nociceptors and therefore provides a measure of acute chemical pain. The second phase (20–60 min) mainly reflects persistent pain that is associated with development of inflammatory response within

the injected paw. Each rat was placed in a transparent Plexiglas box (17.5×23.5×9.5 cm) positioned above a mirror to allow an unobstructed view of the paw. After a 15-min habituation period, formalin (10 μl of a 3% solution in saline) was injected under the plantar surface of the right hind paw, using a 50 μl Hamilton microsyringe with a 25-gauge needle. The rats were placed back into the box immediately after the injection and behavioral scoring consisted of either counting the number of nociceptive responses (paw licking, shaking and biting) or measuring how long the animals produced these responses.

2.4. Monoiodoacetate (MIA)-Induced Osteoarthritis.

A MIA (Sigma-Aldrich, Milan, Italy) injection was used to induce unilateral osteoarthritis into the tibiotarsal joint [3,4]. Briefly, a total of 2% isoflurane was used to anesthetize the rats and the left leg skin was sterilized with 75% ethyl alcohol. The lateral malleolus was found by palpation and a 28-gauge needle was introduced perpendicularly into the cavity between the tibio-fibular and tarsal bone until a distinctive loss of opposition was found. MIA (2 mg/25 μL of saline) was injected monolaterally (ipsilateral paw). Control rats were injected with saline solution.

For the evaluation of the therapeutic effect (acute treatment), MOMAST(®) GR25 was orally administered at doses of 100 mg/kg, 300 mg/kg and 1g/kg on day 14 after MIA injection. Behavioural measurements were performed before and 15, 30, 45 and 60 min after MOMAST(®) GR25 administration. Control animals were treated with vehicle.

To evaluate the protective effect (repeated treatment), MOMAST(®) GR25 was orally administered at doses of 300 mg/kg twice daily starting from the same day of the MIA injection (day 1) and till the end of the experiment (day 14). Behavioural measurements were performed on day 7 and 14 from the beginning of the experiment and were conducted 24 h after the last MOMAST(®) GR25 administration and 30 min following each daily treatment. Control animals were treated with vehicle.

2.5. Paw Pressure Test

Mechanical hyperalgesia was evaluated by an analgesimeter (Ugo Basile, Varese, Italy) according to the methods described by Leighton and collaborators (1988) and Bird and collaborators (2016) [5,6]. Briefly, a regular growing weight was applied to a limited area of the dorsal surface of ipsilateral and contralateral hind paws using a blunt conical probe by a mechanical device. The mechanical pressure (expressed in grams) was increased until vocalization or a withdrawal reflex happened while rats were lightly restrained. A cut-off value of 100 g was adopted.

2.6. Incapacitance Test

Spontaneous pain was evaluated by an incapacitance apparatus (Linton Instrumentation, Norfolk, UK) in order to record variations in the postural equilibrium [7,8]. Rats were trained to stand on their hind paws in a box. This box was located above the incapacitance apparatus, allowing us to autonomously measure the weight that the rat applied on each hind limb. Data represent the mean of five successive measurements for each animal. An unequal distribution of weight on hind limbs showed a monolateral reduced pain threshold [3]. Data were calculated as the difference between the weight applied to the limb contralateral to the injury and the weight applied to the ipsilateral one (Δ weight).

2.7. Statistical analysis

Statistical analysis was performed using GraphPad Prism version 5.01 for Windows (GraphPad Software, San Diego, CA, USA). Means ± SEM were determined for each experimental group and analyzed by one-way analysis of variance (ANOVA), followed by Newman–Keuls comparison multiple test or by Bonferroni test. Statistical significance was set at $p < 0.05$. The number of animals randomized for each experimental group was calculated on the basis of the "Resource Equation" $N = (E + T)/T$ ($10 \leq E \leq 20$) [9], according to the guidelines suggested by the National Centre for the Replacement, Refinement and Reduction of Animals in Research (NC3RS) and reported on the following web site: https://www.nc3rs.org.uk/experimental-designstatistics

References

1. Recinella, L.; Chiavaroli, A.; Orlando, G.; Menghini L.; Ferrante C.; Di Cesare Mannelli L.; Ghelardini, C.; Brunetti, L.; Leone, S. Protective Effects Induced by Two Polyphenolic Liquid Complexes from Olive (Olea europaea, mainly Cultivar Coratina) Pressing Juice in Rat Isolated Tissues Challenged with LPS. *Molecules* **2019**, *24*, 3002.
2. Recinella, L.; Chiavaroli, A.; Di Valerio, V.; Orlando G.; Ferrante, C.; Gesmundo, R. Granata, R. Cai, W. Sha, A.V. Schally, R. Lattanzio, L. Brunetti, S. Leone. Protective effects of growth hormone-releasing hormone analogs in DSS-induced colitis in mice. *Sci. Rep.* **2021**, *11*, 2530.
3. Di Cesare Mannelli, L.; Micheli, L.; Zanardelli, M.; Ghelardini, C. Low dose native type II collagen prevents pain in a rat osteoarthritis model. *BMC Musculoskelet. Disord*, **2013**, *14*, 228.
4. Maresca, M.; Micheli, L.; Cinci, L.; Bilia, A.R.; Ghelardini, C.; Di Cesare Mannelli, L. Pain relieving and protective effects of Astragalus hydroalcoholic extract in rat arthritis models. *J. Pharm. Pharmacol.* **2017**, *69*, 1858–1870.
5. Leighton, G.E.; Rodriguez, R.E.; Hill, R.G.; Hughes, J. κ-Opioid agonist produce antinociception after i.v. and i.c.v. but not intrathecal administration in the rat. *Br. J. Pharmacol.* **1988**, *93*, 553–560.
6. Bird, M.F.; Cerlesi, M.C.; Brown, M.; Malfacini, D.; Vezzi, V.; Molinari, P.; Micheli, L.; Di Cesare Mannelli, L.; Ghelardini, C.; Guerrini, R.; Calò, G.; Lambert, D.G. Characterisation of the novel mixed mu-NOP peptide ligand dermorphin-N/OFQ (DeNo). *PLoS ONE* **2016**, *11*, e0156897.
7. Bove, S.E.; Calcaterra, S.L.; Brooker, R.M.; Huber, C.M.; Guzman, R.E.; Juneau, P.L.; Schrier, D.J.; Kilgore, K.S. Weight bearing as a measure of disease progression and efficacy of anti-inflammatory compounds in a model of monosodium iodoacetate-induced osteoarthritis. *Osteoarthr. Cartil.* **2003**, *11*, 821–830.
8. Maresca, M.; Micheli, L.; Di Cesare Mannelli, L.; Tenci, B.; Innocenti, M.; Khatib, M.; Mulinacci, N.; Ghelardini, C. Acute effect of Capparis spinosa root extracts on rat articular pain. *J. Ethnopharmacol.* **2016**, *193*, 456–465.
9. Charan J., Kantharia N.D. How to calculate sample size in animal studies? *J. Pharmacol. Pharmacother*. **2013**, *4*, 303–306.
